# Examining Sirtuin-1 Levels and Inflammation Markers in Behcet’s Disease: A Pilot Study

**DOI:** 10.5152/ArchRheumatol.2025.11042

**Published:** 2025-06-23

**Authors:** Volkan Ecesoy, Hasan Arıcı, Fadime Pınar Ateş, Usame Ömer Osmanoğlu, Hilal Ecesoy

**Affiliations:** 1 Department of Biochemistry, Karamanoğlu Mehmetbey University, Karaman, Türkiye; 2 Department of Biostatistics, Karamanoğlu Mehmetbey University, Karaman, Türkiye; 3 Department of Physical Therapy and Rehabilitation, Division of Rheumatology, Karamanoğlu Mehmetbey University, Karaman, Türkiye

**Keywords:** Behcet’s disease, inflammation, Sirtuin-1

## Abstract

**Background/Aims:**

Interleukin (IL)-1, IL-6, and tumor necrosis factor (TNF)-α are the principal proinflammatory cytokines that contribute to inflammatory activity in patients with Behcet’s disease (BD). The aim was to assess circulatory Sirtuin-1 (SIRT1) levels in BD and evaluate potential relationships with disease activity scores and several inflammatory markers in order to investigate its potential as a disease activity marker.

**Materials and Methods:**

Forty patients with BD and 40 healthy volunteers matched for age and sex were enrolled. Demographic and clinical data were recorded, including family history of BD, smoking and alcohol consumption, height, weight, and comorbidities. Patients with BD were classified with respect to disease activity, treatments, and organ involvement. Disease activity was determined and categorized using the Behcet Disease Current Activity Form (BDCAF). Inflammation markers and SIRT1 levels were studied in fasting blood samples.

**Results:**

C-reactive protein, IL-6, and TNF-α values were found to be significantly higher in patients with BD compared to controls, largely irrespective of disease activity. SIRT1 results were similar in patients and controls. SIRT1 measurements again showed no differences when compared across disease activity groups and patients with and without TNF-α blockade treatment. However, patients with concurrent vascular and ocular involvement appeared to have significantly lower SIRT1 levels when compared to patients with only ocular involvement.

**Conclusion:**

SIRT1 levels in the circulation of patients with BD appear to be similar to controls, regardless of disease activity or anti-TNF-α treatment; however, SIRT1 concentrations may be associated with vascular injury as demonstrated by significantly lower SIRT1 levels in patients with vascular + ocular involvement compared to those with only skin or ocular involvement.

**Main Points**

- Behçet’s disease is a disease with a wide spectrum of organ involvement.
- There is currently no biomarker that can determine disease activity and severity.
- Low SIRT1 levels may be associated with end-organ damage, particularly when vascular and ocular involvement coexist.

## Introduction

Behcet’s disease (BD) is a variable vascular vasculitis that can affect multiple organ systems. It can cause oral and genital ulcers, papulopustular and nodular skin lesions, arthritis, uveitis, venous and arterial thrombosis, arterial aneurysms, central nervous system lesions, and gastrointestinal ulcers.^1^ The etiopathogenesis of BD remains incompletely understood. As with numerous other connective tissue diseases, the prevailing hypothesis regarding the etiology of BD is that genetically susceptible individuals develop disease manifestations in relation to altered neutrophil and T cell activation, polarization of proinflammatory cytokines, and loss of immune tolerance.^2^

The T-helper (Th)1 immune response is a significant contributor to the pathogenesis of BD, indicating that cytokines secreted as a result of Th1 activation are associated with the activity of BD. Several studies have demonstrated significantly higher levels of Th1 cells, related cytokines, and transcription factors in patients with active BD relative to inactive BD.^3^ Mounting evidence indicates that these cytokines are strong factors in the pathogenesis of BD and may represent promising therapeutic targets.^4,5^ Interleukin (IL)-1, IL-6, and tumor necrosis factor (TNF)-α are the principal proinflammatory cytokines present in patients with BD, which may arise from single nucleotide polymorphisms of these cytokines that increase the expression of these proinflammatory cytokines.^3^ Interleukin-6 is a pleiotropic cytokine produced by innate immune cells and plays a pivotal role in the pathogenesis of BD by influencing the differentiation of CD4+ T cells into Th17 cells. Previous studies have indicated that IL-6 is among the proinflammatory cytokines secreted by mononuclear phagocytes and neutrophils in individuals with BD.^3,4^ Tumor necrosis factor-α is a proinflammatory cytokine that plays a critical role in the induction and sustenance of inflammation. Over the past decade, the use of TNF-α antagonists, including infliximab and adalimumab, has demonstrated efficacy in improving gastrointestinal and joint involvement in BD.^3^

Sirtuins are nicotinamide adenine dinucleotide-dependent class III Deacetylase enzymes and Sirtuin 1 (SIRT1) is expressed in a wide range of mammalian tissues, in both the cytoplasm and the nucleus of cells.^6^ SIRT1 regulates some biological processes, including energy balance, inflammation, oxidative stress, mitochondrial biogenesis, apoptosis, and autophagy. Preclinical and clinical studies have demonstrated the importance of SIRT1 in the pathogenesis of autoimmune diseases, including rheumatoid arthritis, systemic lupus erythematosus, inflammatory bowel disease, multiple sclerosis, and others.^7^

The objective of this study was to ascertain the levels of SIRT1, IL-6, and TNF-α in the serum of patients with BD, to compare them with those of healthy individuals, to elucidate relationships with disease activity scores and organ involvement, and to investigate the potential of SIRT1 as a disease marker for BD.

## Materials and Methods

### Subjects

The study population comprised 40 patients diagnosed with BD in accordance with the International Criteria for Behcet’s Disease^8^ who were followed up and treated at the Karamanoğlu Mehmetbey University Faculty of Medicine Rheumatology Clinic between December 2023 and May 2024. The control group consisted of 40 age- and sex-matched healthy volunteers. Patients who had other autoimmune diseases, infections, or malignancies were excluded from the study. Informed consent was obtained from all subjects. The ethical approval of the study was obtained from the ethics committee of Karamanoğlu Mehmetbey University Medicine Faculty (Approval no: 06-2023/16; 20.06.2023).

### Data Collection

All patients were asked to provide information regarding age, sex, family history of BD, smoking and alcohol consumption, height and weight, additional medical conditions, and medications used for BD. Patients with BD were questioned for the presence of oral aphthae, genital ulcers, erythema nodosum-like lesions, papulopustular lesions, arthritis, arthralgia, uveitis, vascular involvement, neurological involvement, and gastrointestinal involvement. The disease activity status of patients with BD was determined in accordance with the Behcet Disease Current Activity Form (BDCAF).^9^ In accordance with the aforementioned criteria, the patient was assigned 1 point for each of the following symptoms that had manifested within the previous month: headache, oral ulcer, genital ulcer, erythema, skin pustules, arthritis, arthralgia, nausea/vomiting/abdominal pain, diarrhea/rectal bleeding, central nervous system involvement, eye involvement, and major vessel involvement. The total index score was calculated on a scale of 0 to 12. Patients with BD were classified into 2 categories: those with a score of 4 or above were designated as “active disease,” while those with a score below 4 were considered to have “inactive disease.”

Patients were also classified with respect to treatments, including colchicine, TNF-α blockers (infliximab, adalimumab), and immunosuppressive agents (azathioprine, cyclosporine, mycophenolate mofetil), or any combinations of these therapies. Finally, organ involvement was also assessed based on medical records and patient-reported information. The resultant categories and subcategories were also compared with respect to the examined parameters.

### Biochemical Measurements

Peripheral blood samples were collected in a standardized manner from all individuals included in the study. Sera were obtained and stored at −80°C until quantification. The serum levels of IL-6 and TNF-α were determined utilizing a MAGLUMI X8 automatic chemiluminescence immunoassay analyzer (Snibe Diagnostics / PRC). According to the manufacturer, the assay range for IL-6 is 0.5 to 5000 pg/mL, whereas the assay range and limit for TNF-α are 4 to 1000 pg/mL. The normal reference range for IL-6 is ≤7.0 pg/mL, while that for TNF-α is ≤8.0 pg/mL. The serum levels of SIRT1 were determined with a Human SIRT-1 ELISA kit (Sunred Biotechnology Company/PRC) and spectrophotometric measurements were performed using a Varioskan Lux Multimode Microplate Reader (Thermoscientific/USA). In accordance with the manufacturer’s specifications, the assay range for SIRT-1 was 0.5 to 40 ng/mL, with a sensitivity of 0.306 ng/mL.

### Statistics

Statistical analyses were performed using the IBM Statistical Package for Social Sciences 25.0 (IBM SPSS Corp.; Armonk, NY, USA). Correlation heatmaps were generated using Python 3.7.9 (PSF; Delaware, USA) software. Numerical variable distributions were assessed for normal distribution using the Shapiro-Wilk test and homogeneity of variance was tested with the Levene test. Based on the results, the Student *t*-test, the Mann-Whitney *U* test, the Kruskal-Wallis test, and the Spearman Correlation Analysis were used for analyses between groups. Multiple testing corrections were applied when necessary, and pairwise significances were summarized with the letter notation approach when the primary *P* value was significant. In this approach, groups with similar values are labeled with the same letter, which results in a simplified demonstration of significant pairwise differences. For example, “a,” “b,” and “ab” in different columns of the same row indicate that the first 2 groups are significantly different, while the third group is similar to both the first and second groups. A lack of labels indicates that either the primary *P* value is non-significant, or the unlabeled group is significantly different from all other groups. Continuous data were presented as mean ± SD and median (Q1-Q3; interquartile range, IQR). The statistical significance level was defined as an alpha value of 5% (*P* < .05). When assessing correlation coefficients, the relationships were defined as weak (0-0.300 coefficient), moderate (0.301-0.500), and strong (>0.500). Statistically significant values are marked in bold.

## Results

The study cohort comprised 40 patients with BD (19 females, 21 males) and 40 age-and sex-matched healthy controls (21 females, 19 males). As anticipated, C-reactive protein (CRP), IL-6, and TNF-α values were found to be significantly higher in patients with BD relative to controls. The SIRT1 values were observed to be higher in the patient group than in the control group; however, no statistical significance was found (Table 1).

As determined by BDCAF, 12 patients were identified as having active disease, while 28 were classified as having inactive disease. These 2 patient groups were similar in terms of CRP and IL-6 levels, while TNF-α was significantly higher in patients with active disease compared to the inactive group. Both the active and inactive disease groups had significantly higher TNF-α compared to controls. SIRT1 values were again similar in patients with active and inactive disease (Table 2).

Ten patients were receiving colchicine exclusively, 11 were treated with TNF-α blockers (8 with infliximab, 3 with adalimumab), and 18 were prescribed immunosuppressive agents (azathioprine, cyclosporine, mycophenolate mofetil). One patient was not receiving treatment at recruitment due to a new diagnosis. When examined for treatment groups, it was found that recipients of TNF-α blockade had significantly higher TNF-α levels compared to non-recipients. No significant differences were observed in the levels of SIRT1 and IL-6 when examined for treatments (Table 3).

Organ involvement analyses revealed that TNF-α was significantly higher in those with vascular + ocular involvement compared to those with only skin involvement. Interestingly, SIRT1 was found to be significantly lower in those with vascular + ocular involvement compared to those with only ocular and those with only skin involvement (Table 4).

**Figure 1.**
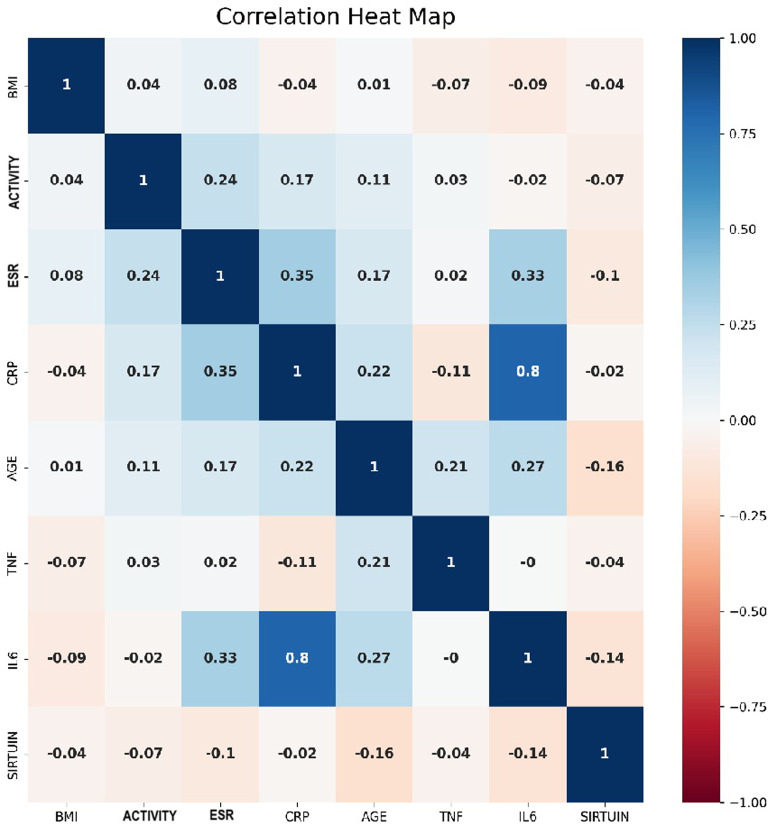
Correlation heat map of variables.

The analysis of directional relationships between quantitative variables in the BD group yielded a weak positive relationship between activity status and age, as well as between activity status and CRP and erythrocyte sedimentation rate (ESR). Additionally, IL-6 had a weak positive relationship with age, a moderate positive relationship with ESR, and a strong positive relationship with CRP. Negligible negative correlations were observed between SIRT1 and other variables included in the correlation analysis (Figure 1).

## Discussion

Türkiye is a country with a high prevalence of BD. Early diagnosis, activity assessment, and appropriate management are crucial to prevent morbidities (especially blindness) and risk of mortality (often through pulmonary artery or brain involvement). Although tools are available to assess disease activity (such as the BDCAF), the accuracy of these methods and their overall utility remain a topic of debate since these rely upon clinical assessment and experience. Biochemical markers that can provide a swift assessment of disease activity and severity would considerably enhance the management of patients. The goal was to assess SIRT1 levels and determine whether it was associated with inflammatory activity markers such as IL-6 and TNF-α in patients with BD. To the authors’ knowledge, SIRT1 has been quantified in only 1 study enrolling BD patients with ocular involvement.^10^ The authors studied SIRT1 in T cells obtained from anticoagulated blood samples. Despite the lack of significant differences in the BD vs. controls and active disease vs. inactive disease comparisons, it was interestingly found that patients with only ocular involvement had significantly higher SIRT1 levels when compared to those with vascular + ocular involvement. A similar difference was observed for skin involvement; however, this result was not considered relevant to interpret due to the extremely small size of the skin involvement group (n = 3). Interestingly, patients with vascular + ocular involvement also had the highest TNF-α levels in this comparison, suggesting that SIRT1 levels could be correlated negatively with TNF-α levels (and perhaps inflammatory activity) in patients with vascular + ocular involvement. No advanced statistical analyses were performed in this subgroup due to the limited patient count (n = 9); however, future studies may benefit from analyzing this possible relationship with larger patient groups.

The results of studies on SIRT1 levels in autoimmune diseases have been found to vary greatly. In some studies, SIRT1 levels were observed to be elevated in the patient cohort relative to controls or in active patients compared to those with inactive disease.^11,12^ In contrast, others have found lower SIRT1 in patients with autoimmune/inflammatory diseases relative to healthy controls.^13,14^ The correlations between SIRT1 and disease markers also vary depending on the specific disease and study in question. Tan et al^15^ identified a positive correlation between SIRT1 and disease activation markers in patients with rheumatoid arthritis, indicating that SIRT1 may be associated with the proinflammatory status and its levels could fluctuate either as a result or cause of worse disease prognosis in rheumatoid arthritis.^14,15^ However, Wu and colleagues observed that SIRT1 activity was diminished in the peripheral blood of rheumatoid arthritis patients relative to healthy controls. Furthermore, they noted a negative correlation between SIRT1 activity and TNF-α, NF-kB, and other markers, which they interpreted as evidence that SIRT1 exerts an anti-inflammatory effect in rheumatoid arthritis.^16^ The results suggest that SIRT1 levels are unassociated with BD presence or its activity but may be associated with vascular injury, particularly in the context of chronic complications such as thrombosis, aneurysms, or ischemia, as demonstrated by significantly lower SIRT1 levels in patients with vascular + ocular involvement. Furthermore, although the current study is limited in size to perform correlation analyses within the vascular + ocular subgroup, it appears that the results also suggest a negative correlation between SIRT1 and inflammation in this specific subgroup, possibly in relation to vascular involvement. Nonetheless, the present data can only be interpreted to recommend the conduct of larger studies examining whether inflammatory activity correlates negatively with SIRT1 in BD patients with multiple-organ or vascular involvement.

Despite extremely low correlation coefficients, the results showed that SIRT1 levels always appeared to have negative relationships with other inflammatory markers and disease activity. The lack of statistical significance may be attributed to the limited sample size and the fact that most of the patients were undergoing treatment. Shen et al^7^ observed that the hyperacetylation of NF-κB p65 and elevated levels of proinflammatory cytokines, including TNF-α and IL-1β, were present in SIRT1-deficient macrophages in comparison to their controls. The present study revealed elevated levels of TNF-α, particularly in patients undergoing TNF-α blockade. A review of the medical history of these patients revealed that these subjects had been started on anti-TNF-α therapies due to a history of highly active disease, including recurrent panuveitis, pulmonary artery involvement, and central nervous system involvement, which did not respond to other immunosuppressive treatment. It is plausible that the elevated TNF-α levels observed in this study were a consequence of the timing of the blood collection. The blood samples were obtained immediately prior to the administration of the treatment dose, which occurred at the outpatient clinic when the patient presented for TNF-α treatment.

There is insufficient information on how SIRT1 values are affected by immunosuppressive treatment. A study in rats showed that SIRT1 protein levels increased as a result of azathioprine treatment of rat hearts in vitro.^17^ In a study conducted on patients with multiple sclerosis, a significant decrease in SIRT1 levels was found in patients treated with interferon-β compared to controls, while no change was found in those treated with fingolimod.^18^ The findings appear to support the suggestion that immunosuppressive treatments have limited impacts on SIRT1 levels, especially since TNF-α blockade was found to be the only treatment that resulted in significant differences in terms of inflammation markers.

Sirtuins are NAD+-dependent histone deacetylases that play a role in the pathophysiology of cardiovascular diseases and have been identified as important regulators of endothelial cell function. A few recent studies suggest that an imbalance in the regulation of endothelial sirtuins, particularly SIRT1, contributes to endothelial cell dysfunction.^19^ Manetti et al^20^ found low SIRT1 levels in patients with systemic sclerosis who had vascular damage. As mentioned previously, this study indicates that SIRT1 levels are significantly lower in patients with vascular + ocular involvement relative to those with only ocular involvement. Although this could suggest alterations related to multiple organ involvement, it could also be a result of the vascular aspect. Further research is definitely necessary to support this hypothesis; however, this clear difference in comparative results suggests that circulatory SIRT1 may be lowered as a result of vascular involvement in patients with BD.

### Limitations

The present study is limited by its single-center design, relatively small sample size, and inclusion of patients undergoing treatment. Although the authors were selective in applying analyses only when statistical assumptions were met, it is crucial to note that researchers should be cautious when interpreting results drawn from analyses of groups with small sample sizes. Also, this study was conducted with a single blood sample taken from patients and does assess longitudinal changes or alterations in response to treatment over time. In relation to this, steroid use or its dosage was also not examined in the present study. This is an important factor that has considerable impacts on inflammatory activity and systemic metabolism, which might bias the measurements and the relationships observed in the analyses. Finally, although no strong conclusions based on the correlation analyses were arrived upon, it must be mentioned that SIRT1 did not demonstrate any notable relationships when examined in the whole group. However, these limited relationships could prove to be important in subgroup analyses (such as those with multiple-organ or vascular involvement). Thus, ongoing research on this topic should aim to include much larger group sizes to accommodate for more reliable subgroup analyses.

The data shows that SIRT1 levels were similar to healthy controls in the group of patients with BD. Also, disease activity and type of treatment appeared to have no impact on SIRT1 levels. However, a detailed subgroup analysis showed that BD patients with vascular + ocular involvement had significantly lower SIRT1 levels when compared to those with only ocular involvement, suggesting that SIRT1 could be associated with either multiple organ damage or vascular involvement in patients with BD.

## Figures and Tables

**Table 1.** Comparison of the Behcet’s Disease and Control Groups in Terms of Examined Parameters

Parameter	Behcet’s Disease			Control			*P*
	n	Mean ± SD	Median (Q1-Q3)	n	Mean ± SD	Median (Q1-Q3)	
Age (years)	40	40.33 ± 8.42	41.00 (34.00-45.75)	40	39.63 ± 8.57	43.00 (33.25-46.00)	.996
ESR (mm/h)	11	9.75 ± 13.20	4.50 (4.00-10.75)	40	6.91 ± 12.69	6.00 (4.00-11.00)	.684
CRP (mg/L)	40	5.63 ± 7.43	2.95 (1.33-6.56)	40	1.52 ± 1.13	1.20 (0.60-2.08)	**<.001**
BMI (kg/m^2^)	40	26.06 ± 4.59	25.40 (23.86-27.31)	40	25.44 ± 3.60	25.20 (22.90-28.71)	.814
IL-6 (pg/mL)	40	5.03 ± 5.70	3.93 (2.02-5.23)	40	2.08 ± 0.97	1.93 (1.28-2.83)	**<.001**
TNF-α (pg/mL)	40	33.87 ± 65.20	9.87 (5.07-19.58)	40	6.38 ± 4.27	5.04 (3.91-7.41)	**<.001**
SIRT1 (ng/mL)	40	10.02 ± 8.93	7.11 (4.19-13.79)	40	9.85 ± 8.70	6.63 (3.47-15.82)	.740

Mann–Whitney *U* Test was applied.

BMI, body mass index; CRP, C-reactive protein; ESR, erythrocyte sedimentation rate; IL-6, interleukin-6; n, sample size; Q1, 1st quartile; Q2, 2nd quartile; TNF-α, tumor necrosis factor-α.

**Table 2.** Comparison of the Results Between Controls and Patients with Active and Inactive Disease

Parameter	Control			Inactive BD			Active BD			*P*
	n	Mean ± SD	Median (Q1-Q3)	n	Mean ± SD	Median (Q1-Q3)	n	Mean ± SD	Median (Q1-Q3)	
ESR (mm/h)	11	6.91 ± 3.56	6.00 (4.00-11.00)	28	8.86 ± 11.85	4.00 (4.00-9.75)	12	11.83 ± 16.33	6.00 (4.00-11.75)	.520*
CRP (mg/L)	40	1.52 ± 1.13^a^	1.20 (0.60-2.08)	28	5.60 ± 8.09^b^	3.30 (1.38-6.40)	12	5.70 ± 5.90^b^	2.50 (1.33-10.35)	<.001*
IL-6 (pg/mL)	40	2.08 ± 0.97^a^	1.93 (1.28-2.83)	28	5.54 ± 6.73^b^	3.93 (1.73-6.98)	12	3.83 ± 1.42^b^	4.01 (2.42-5.23)	<.001*
TNF-α (pg/mL)	40	6.38 ± 4.27^a^	5.04 (3.91-7.41)	28	28.57 ± 46.72^b^	10.30 (5.12-19.58)	12	46.24 ± 97.36^ab^	7.46 (4.98-20.90)	.001*
SIRT1 (ng/mL)	40	9.85 ± 8.70	6.63 (3.47-15.82)	28	11.22 ± 10.28	6.51 (4.19-16.97)	12	7.22 ± 3.33	7.81 (3.73-10.19)	.791*

BD, Behcet’s Disease; CRP, C-reactive protein; ESR, erythrocyte sedimentation rate; IL-6, interleukin-6; n, sample size; Q1, 1st quartile; Q2, 2nd quartile; TNF-α, tumor necrosis factor-α.

*Kruskal–Wallis Test was applied.

^a^ and ^b^ indicate pairwise differences between groups. Any measurements with shared superscript letters are not significantly different from each other.

**Table 3.** Descriptive Statistics of the Parameters by Biological Agent Usage

Parameter	Using TNF-α Blockade			Not Using TNF-α Blockade			*P*
	n	Mean ± SD	Median (Q1-Q3)	n	Mean ± SD	Median (Q1-Q3)	
ESR (mm/h)	11	10.27 ± 16.32	4.00 (4.00-9.00)	29	9.55 ± 12.15	5.00 (4.00-11.00)	.654*
CRP (mg/L)	11	3.83 ± 4.28	1.90 (1.00-6.10)	29	6.32 ± 8.28	3.60 (1.65-7.45)	.175*
IL6 (pg/mL)	11	5.27 ± 3.34	4.92 (1.77-8.17)	29	4.94 ± 6.43	3.62 (2.11-4.61)	.241*
TNF-α (pg/mL)	11	103.25 ± 96.14	64.00 (23.80-152.00)	29	7.56 ± 3.51	7.44 (4.67-10.30)	<.001*
SIRT1 (ng/mL)	11	7.64 ± 4.39	8.35 (4.25-10.47)	29	10.92 ± 10.06	6.47 (3.82-16.93)	.765*

CRP, C-reactive protein; ESR, erythrocyte sedimentation rate; IL-6, interleukin-6; n, sample size; Q1, 1st quartile; Q2, 2nd quartile; TNF-α, tumor necrosis factor-α.

*Mann–Whitney *U* Test was applied.

**Table 4.** Comparison of Organ Involvement Groups

Parameter	Skin			Ocular			Vascular + Ocular			*P*
	n	Mean ± SD	Median (Q1-Q3)	n	Mean ± SD	Median (Q1-Q3)	n	Mean ± SD	Median (Q1-Q3)	
Age (year)	3	36.00 ± 5.00	36.00 (31.00-.)	28	41.25 ± 9.56	42.50 (32.00-48.00)	9	38.89 ± 4.31	38.00 (35.00-42.00)	.376
ESR (mm/h)	3	25.67 ± 31.66	11.00 (4.00-.)	28	7.68 ± 6.89	5.00 (4.00-10.00)	9	10.89 ± 18.09	4.00 (4.00-10.00)	.354
CRP (mg/L)	3	4.53 ± 5.22	2.30 (8.00-.)	28	6.29 ± 8.46	3.30 (1.30-7.88)	9	3.94 ± 3.92	2.20 (1.65-5.25)	.794
IL6 (pg/ml)	3	2.53 ± 1.60	1.80 (1.43-.)	28	5.21 ± 6.51	3.76 (2.32-5.23)	9	5.30 ± 3.52	4.19 (2.20-8.46)	.374
TNF-α (pg/ml)	3	3.53 ± 0.70^a^	3.58 (2.84-.)	28	31.97 ± 71.90^ab^	9.26 (6.17-12.43)	9	49.90 ± 51.31^b^	44.20 (7.89-85.50)	**.013**
SIRT1 (ng/ml)	3	12.59 ± 9.32^a^	11.00 (4.17-.)	28	11.66 ± 9.61^a^	8.91 (4.90-16.54)	9	4.05 ± 1.85^b^	4.25 (3.11-5.57)	**.019**

Kruskal–Wallis test was applied.

n, sample size; Q1, 1st quartile; Q2, 2nd quartile.

^a^ and ^b^ indicate pairwise differences between groups. Any measurements with shared superscript letters are not significantly different from each other.

## Data Availability

The data that support the findings of this study are available on request from the corresponding author.
